# Understanding Occupational Therapists' Job Satisfaction Through an Ecological Lens—A Qualitative Scoping Review

**DOI:** 10.1155/oti/3268526

**Published:** 2025-03-19

**Authors:** Brodie Dupre, Nasim Salehi

**Affiliations:** ^1^Faculty of Health, Southern Cross University, Bilinga, Queensland, Australia; ^2^Faculty of Health Sciences and Medicine, Bond University, Gold Coast, Queensland, Australia

**Keywords:** job satisfaction, occupational therapy, professional development, professional identity

## Abstract

Despite the critical role of occupational therapists in healthcare, there has been limited focus on understanding their job satisfaction compared to other health-related disciplines. This gap is significant as job satisfaction among occupational therapists profoundly influences employee morale, client care, and organizational dynamics. This study explored factors influencing occupational therapists' job satisfaction through an ecological lens to enhance their work environments, promoting both personal and professional growth. A scoping review of qualitative evidence was conducted from 1921 to 2024 via CINAHL, Medline, Scopus, and AMED, including 10 qualitative articles. Thematic analysis was used to identify key themes. Four key themes were identified impacting job satisfaction including humanistic values driving professional fulfillment, professional identity and recognition, workplace structural barriers, and advocacy and strengthening approaches. Although occupational therapists find their job rewarding and fulfilling because of its client-centered care, they confront challenges particularly with professional recognition, career identity, and limited support. To enhance their job satisfaction, a more system-level ecological approach is required to enhance the leadership approaches, mentorship, communication channels, and collaboration opportunities.

## 1. Introduction

Job satisfaction is a well-known phenomenon that is multifaceted, with contributing intrinsic and extrinsic factors. It is defined as being content with work-related activities and the work environment. Based on Herzberg's theory [1], positive and negative aspects of working environments can be classified under motivation (intrinsic) and hygiene (extrinsic). Motivation relates to the intrinsic aspect such as working responsibilities and recognition (feeling valued). While hygiene factors relate to the “context” of the job, including environment, administration, and policy, these are more extrinsic [2]. Overall, work satisfaction can be less tangible due to the subjectivity of these internal factors in comparison to the working environment, which could make it quite complex to address. Job satisfaction is a key indicator of the psychosocial work environment [3], resulting in a safe and positive working atmosphere that can impact the quality of care provided to consumers [4]. Job satisfaction has a strong correlation with retention, productivity, employee health and wellbeing, and positive consumer outcomes [3, 5].

There is a rise in healthcare turnover, as it was shown that in 2016 the healthcare field turnover was second highest among all industries after hospitality in the United States [5]. Within healthcare, occupational therapists work in various settings, although recruiting and retaining a skilled workforce remain a key challenge [6]. Low retention of unsatisfied occupational therapists' results in loss of “institutional knowledge” and can have a “contagion effect” on the team [7]. Hence, a critical issue for healthcare leaders and organizations is safeguarding staff retention, as high turnover creates a vicious cycle, risking quality of care and resulting in financial complications [5].

Although literature showed that occupational therapists are generally satisfied, there is no clear consensus about the *level* of job satisfaction within their roles. A frequently cited study within this field is Davis and Bordeiri [2], who used Herzberg's theory [1] to conduct a questionnaire of 249 American occupational therapists in relation to job satisfaction and autonomy, indicating moderate to high job satisfaction. Taylor et al., [8] surveyed 110 occupational therapists in Canada using the Minnesota Job Satisfaction Questionnaire and found no statistical difference in job satisfaction between genders. Eklund and Hallberg [3] surveyed a large sample of 334 Swedish occupational therapists working in psychiatric care, indicating relatively high job satisfaction. Scanlan and Still [9] conducted a large survey of 110 Australian mental health clinicians, of which 34 were occupational therapists, highlighting high job satisfaction. Sewpersadh and Govender [10] conducted a cross-sectional study of 95 occupational therapists working in a range of settings in South Africa and determined that they had ambivalent feelings about their roles, with the highest satisfaction related to the nature of work, work conditions, and working with others.

Although there are some quantitative reviews measuring the job satisfaction of occupational therapists, no existing critical review was identified which synthesizes the qualitative evidence exploring job satisfaction and the factors affecting this phenomenon. In this review, the aim was to focus on qualitative studies to explore an in-depth understanding of job satisfaction, through the perspectives and narrative of the participants to support their career and work environment. This qualitative review compliments the previous quantitative reviews such as the study conducted by Mertala et al. [11], whose systematic review examines the factors impacting occupational therapists' job satisfaction and their work wellbeing. The study highlighted that although there was a general high job satisfaction, there were mixed findings about career identity, burnout, and social environment. The other older systematic reviews identified focused on stress and burnout of occupational therapists and difficulty with retaining more senior staff [12]. This review specifically focused on occupational therapy mental health clinicians and how to effectively manage stress by identifying moderators of stress in mental health occupational therapists.

The PICo (population, phenomenon of interest, context) format was used to guide this review's question structure [13] and searching strategy [13]. The aim of this review was to advance the understanding of occupational therapists' perceptions surrounding job satisfaction to enhance the policy and practices around their job satisfaction related to both intrinsic and extrinsic factors [13]. Comprehensive understanding of the key factors impacting the experience of occupational therapists' job satisfaction is necessary to empower them personally and professionally. This eventually enhances workplace environments, with substantial gains across different levels/partners, including employees, consumers, organizations, and the community [2].

## 2. Method

### 2.1. Search Strategy and Research Questions

In this study, we utilized a scoping review methodology to explore the experience of job satisfaction of occupational therapists. Scoping reviews can assist in providing a considerable summary of diverse relevant literature, as well as identifying research gaps, particularly when there is scattered information around a topic, although the quality of papers is not assessed. Scoping reviews help with setting research agendas and enhancing evidence-based practices by provision of recommendations to policymakers. We adopted the “PRISMA-ScR extension,” which includes a comprehensive checklist in line with best practices for conducting scoping reviews. This approach ensures transparency and high quality, as detailed by Tricco et al. [14]. The framework included identifying the research question; identifying search strategy; selecting the studies; charting the data, and collating, summarizing, and reporting the results.

Multiple brainstorming meetings were organized around the research question. Coauthors have expertise in occupational therapy, healthcare leadership, and health promotion, providing opportunity to look at the research more holistically. The proposed research question was *what are the key factors influencing the experience of job satisfaction of occupational therapists, working in health, social, and community care settings?*

The authors arranged multiple meetings (with input from librarians) to arrive at a consensus about the keywords related to the research question for each database and develop the searching strategy. Four databases CINAHL, Medline, AMED, and Scopus were searched for publications from 1921 (when occupational therapy became a profession) to 2024. CINAHL, Medline, and AMED databases were chosen as they are specific to allied health and biomedicine therefore anticipated to yield more relevant literature [15]. Scopus was chosen due to its size and access to peer-reviewed articles. Despite relevance to the population, OTseeker typically contains systematic reviews or randomized control trials, therefore was not suitable to search.

The search strategy used a combination of subject headings and free-text keywords (see Table 1). Aiming to balance the search sensitivity and specificity [16], the following key search terms and synonyms were selected and combined with Boolean operators: occupational therapy, occupational therapist, OT; and job satisfaction, work satisfaction, employee satisfaction; and determinant, factor, influence. Selecting the title field descriptor and truncation were additional search strategies employed to ensure articles relevant to the research question were obtained [15]. Delimiters of English language and scholarly articles were used across all databases to gather primary articles of the highest quality. Additionally, a delimiter of healthcare sector was included in Scopus to refine the search results to more relevant articles. Secondary and grey sources of literature were excluded from this review.

### 2.2. Inclusion and Exclusion Criteria for Study Selection

The following inclusion and exclusion criteria were applied during article selection.

Inclusion criteria:

- Original qualitative or mixed-method study designs (with the focus on qualitative phase) exploring the perspectives of occupational therapists on job satisfaction
- Studies which investigated job satisfaction or a combination of factors including job satisfaction
- Occupational therapists working in a healthcare setting nationally and internationally
- Studies published in English language, as resources were limited for translation [17]
- Scholarly (peer-reviewed) studies

Exclusion criteria:

- Quantitative designs
- Studies which researched other allied health professionals or occupational therapy students
- Studies which focused on a specific occupational therapy professional population such as male occupational therapists or new graduates, as these were not considered representative of the perspectives of the wider occupational therapy profession

**Figure 1:**
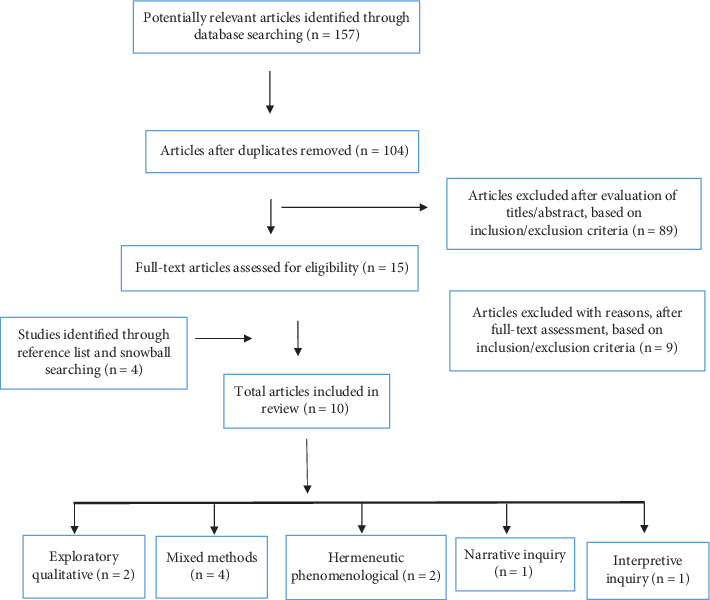
Modified PRISMA flow diagram of article screening and selection.

The search strategy, including the databases, key search terms, and the number of articles found using this method, is shown in Table 1. The search was undertaken during February 2024, resulting in a total of 157 articles. Once duplicates (*n* = 53 articles) were removed, the titles of 104 articles were screened, with 36 article abstracts screened for inclusion in this review, as outlined in Figure 1. Most of the total rejected articles (*n* = 89) were excluded for the following reasons:

- Study designs were not suited to a qualitative review such as Eklund and Hallberg [3] and Mason and Hennigan [5], who both used a quantitative survey to measure job satisfaction therefore not gaining the qualitative perspectives of occupational therapists as intended for this study
- Studies did not include all PICo key terms and therefore the research outcome was unrelated such as Harris [18], who researched the meaning of craft in occupational therapy
- Research articles had too narrow of a population such as Brown [19] who focused solely on the experience of male occupational therapists

The remaining 15 full-text articles were screened for inclusion in this review. A further six articles were excluded for the following reasons:

- The phenomenon of interest and participants did not match the PICo key terms; therefore, outcomes were not relevant [17], such as Haertl al. [20] who studied the influencing factors on satisfaction and efficacy of the occupational therapy service instead of occupational therapists' employment satisfaction
- Articles which used solely quantitative data therefore did not meet study design inclusion criteria as in Sewpersadh et al. [10], Scanlan et al. [9], and Scanlan et al. [7]
- Wills and Case-Smith [21] studied the perception of occupational therapists' role in a rural school; therefore, the research setting and phenomenon of interest did not meet inclusion criteria

Citation chaining occurred in the remaining nine articles which resulted in four eligible new publications. Abstract reviews determined three were not suitable given they were quantitative; however, one was deemed eligible and was included in this review [22].

### 2.3. Charting, Summarizing, and Reporting the Results

The findings were analyzed using the framework suggested by Braun and Clarke [23]. Thematic analysis was used as part of the process of narrative synthesis. In the first step of Braun and Clarke's approach (familiarization with the data), one of the authors (B.D.) extracted the data, and papers were cross-checked by both authors to decrease bias and improve consistency. A data extraction table was developed, including the name of authors (year), country, aims, study design (qualitative research approach), sample/participants, data collection and analysis, results (key findings/themes), and limitations, as shown in Table 2. In the second step, preliminary codes and categories were identified by both authors. The categories were discussed multiple times by both the authors to achieve consensus around the final key themes. In the third step, searching for themes, the first author organized categories into themes and discussed them with the other author. The themes were reviewed, labeled, and defined by both authors.

## 3. Results

### 3.1. Study Characteristics

There was a total of 10 papers that met the inclusion criteria for this review and specifically focused on in-depth exploration of occupational therapists' job satisfaction. Studies had various research designs, including hermeneutic phenomenological studies (*n* = 2), exploratory qualitative studies (*n* = 2), interpretive inquiry study (*n* = 1), narrative inquiry (*n* = 1), and mixed-method study (*n* = 4), where only the qualitative phase was included. Studies were conducted in various countries, including Australia (*n* = 6), New Zealand (*n* = 1), Jordan (*n* = 1), Slovenia (*n* = 1), and Denmark (*n* = 1). The oldest study was published in 1992, and the most recent one was published in 2021. Studies were conducted in various settings such as acute hospitals (*n* = 4), mental health (*n* = 2), a mix of acute hospital and community (*n* = 2), and a mix of diverse range of settings, including hospitals (*n* = 2). Participants were female dominated and ranged from 6 to 93 across different studies. The participants' clinical experience varied from 6 months to 35 years, although four of the studies were mainly dominated by junior therapists (e.g., 18 of the 20 participants in Shiri [33] had 1–3 years of clinical experience; 11 of the 26 participants in Wesley and Clemson [25] were less than 25 years old; 70% of participants in Hayes et al. [27] had 6 or less years of experience; and 85% of participants in Tariah et al. [34] had less than 5 years' experience). Five out of the 10 studies adopted a general semistructured interview to achieve a descriptive picture, allowing participants to express their views in a flexible way. Some of the studies, including Moore et al. [31] and Wesley and Clemson [25] used specific theoretical approaches, such as Herzberg's theory [1] to direct data collection. Three of the studies used focus groups [25, 32, 33], and the last two studies used disseminated surveys [27, 34].

**Figure 2:**
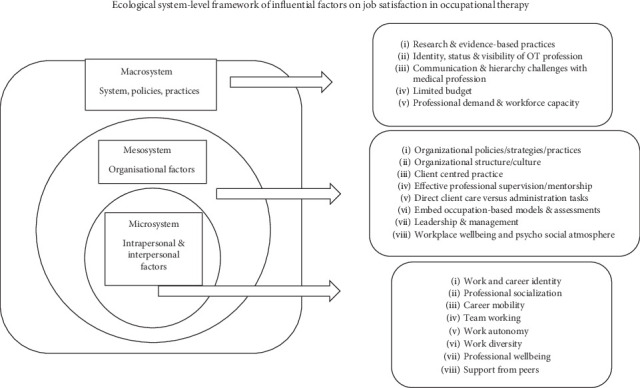
Ecological system-level framework of influential factors on job satisfaction in occupational therapy.

Four key themes were identified in this review, using narrative thematic analysis that significantly impact the job satisfaction of occupational therapists including humanistic values driving professional fulfillment, professional identity and recognition, workplace structural barriers, and advocacy and strengthening approaches. These themes were identified through a comprehensive analysis of various factors considering an ecological lens, including personal, organizational, and systemic factors that influence occupational therapists' professional experiences and wellbeing, as summarized in Figure 2.

### 3.2. Humanistic Values Driving Professional Fulfillment

In almost all the studies, occupational therapists highlighted the specific nature of their occupation that gives them meaning and purpose beyond just a job resulting in high level of satisfaction. They feel their job is fulfilling and rewarding, due to its humanistic approach, looking at clients' health and wellbeing in a comprehensive and functional way, not only the physical aspect but also the mental, psychological, and social aspects. This holistic and client-centered approach empowers individuals to take control of their health, wellbeing, and independence, by valuing them and listening to them as needed to make positive connections. Occupational therapists feel they have a higher contribution to the community by empowering clients about their health and wellbeing through their unique lens of occupational performance.

Client-centered care was integral to the principles and core values within occupational therapy, enhancing autonomy and creativity. Although occupational therapists highly regard the humanistic and client-centered nature of the role, they acknowledge that this can be both challenging and rewarding [22, 25, 26, 31, 34]. One of the key challenges raised was when client interaction is limited, due to an imbalance between direct client care and administration tasks, which requires higher levels of workload and negatively effects job satisfaction [24]. Occupational therapists suggested that to be able to make those positive connections with clients, some characteristics and traits are required at the personal level or interpersonal characteristics. Key characteristics include sustaining professional resilience, positive attitudes, and identity, particularly in specific settings such as mental health [22]. The other traits included adaptability, creativity [32], and autonomy [31]. Additionally, empathy, communication skills, the passion to connect with clients, and making the intervention personalized were highlighted as key traits [34]. These characteristics likely enable occupational therapists to effectively interact with their clients, navigate challenging work environments, and derive fulfillment from their profession.

The concept of autonomy is emphasized as contributing to fulfillment and impacting the quality-of-care provision, job satisfaction, and identity [24, 25, 31]. The type of autonomy includes job variety and flexibility, which allows for a personalized approach to care provision rather than a one-size-fits-all approach. Examples include the ability to independently plan their interventions for clients [31], make their own clinical decisions [24], and set their own goals [25] that overall will enhance occupational therapists' freedom, confidence, and control over their work.

Creativity is another core aspect of meaningful job engagement highlighted by participants that is interconnected with autonomy and creating an enjoyable challenge for occupational therapists to provide personalized care. There can be various factors that foster creativity among occupational therapists, including the uniqueness of each client case [32]. Other personal traits, such as enthusiasm and curiosity for learning and developing, are critical in creating a creative work environment. More experienced occupational therapists tend to find it easier to apply creative solutions, gaining from their broad knowledge and skills. The work environment also plays a crucial role in supporting and stimulating the setting for encouraging creativity. Positive and supportive relationships with colleagues and superiors enhance creativity by promoting an atmosphere of trust and collaboration. Ultimately, creativity is a key factor in job satisfaction among occupational therapists, empowering them to have freedom and engage in creative roles providing a significant sense of accomplishment and fulfillment [32].

### 3.3. Professional Identity and Recognition

The occupational therapy profession frequently struggles with professional identity and recognition challenges from other disciplines, as well as the public. Examples are misunderstandings about their role and contribution to clients' health resulting in frustration and the underutilization of their skills [22, 31, 34]. Hence, this can lead to having pressure to conform to more medically rigorous disciplines, conflicting with the holistic, client-centered approach that is the core to occupational therapy, which potentially compromises their working values and identity [22].

In environments with limited interdisciplinary collaboration and recognition, occupational therapists may feel isolated, impacting their professional growth, job clarity, and satisfaction [33]. Challenges in maintaining professional and career identity are particularly obvious in specialized settings such as eating disorder units or mental health departments [22, 26].

The occupational therapy role is often poorly understood by the public and other healthcare professionals, especially doctors, which can lead to role misconceptions and misinterpretations resulting in frustrations in their meaningful roles and contribution [33, 34]. These issues related to career identity, coupled with the high compassion requirements of the role, can contribute to occupational burnout and feelings of being undervalued [26, 28, 31]. Moreover, the traditional healthcare system's hierarchy and bureaucracy further aggravate disparities in perceived professional status, negatively impacting professional identity, communication, and the quality of referrals [25, 33, 34].

Occupational therapists have a responsibility to express the efficacy of using occupation as a therapeutic modality more clearly to avoid being undervalued or overly influenced by other disciplines [22]. Employing occupation-based models, defining their roles clearly, more meaningful integration with other disciplines, and leadership and managerial support can all enhance occupational therapists' contributions and recognition within multidisciplinary settings improving their professional resilience and job satisfaction [22, 26–28, 31, 33].

### 3.4. Workplace Structural Barriers

Workplace structural barriers impact job satisfaction including limited support and resources, management and leadership approaches, working design, collaboration, and new structures such as the new public management (NPM). Inadequate organizational support and limited resources such as staffing, materials, and financial support significantly impact the effectiveness of quality care provision and contribute to pressure and stressful working environments. Occupational therapists may feel their ability to provide effective care is compromised by these limitations [25, 31]. Similarly, occupational therapists highlighted workplace-induced stress and burnout, including high job demands such as substantial workloads and the intense emotional involvement required in patient care, can lead to burnout particularly in specific settings of the healthcare practice such as eating disorder units [26]. In addition, in mental health settings, there is inadequate recovery time for occupational therapists to reset between sessions, contributing to professional fatigue and chronic occupational stress [27]. Furthermore, the role of managers and leaders was emphasized as they often do not provide sufficient appropriate support [28, 31].

Balancing the extensive client demand with the available workforce was frequently identified as a source of dissatisfaction [24, 31]. Budget limitations could impact the professional growth of occupational therapists and result in destructive competition between the team members that could potentially impact team cohesion [25, 33]. Strategies to improve this situation can be restructuring the profession by clear definition of the role [33], improving the quality of training programs [34], and access to more practical evidence-based practice through strengthening the research field, as well as promoting the profession [33] to validate their practice [22, 31].

An example can be structural approaches integrating occupational therapists with other allied health disciplines, which can be beneficial for integrated care but require precise planning that prevent workplace stressors and rather enhance a collaborative, smooth integration [24]. In the case of working with other disciplines, occupational therapists emphasized the importance of understanding their unique role and contribution. For example, often there can be challenges occupational therapists face in various settings, particularly acute care, where there is a significant lack of understanding about the occupational therapy role by other healthcare professionals, especially doctors [33]. This misunderstanding can lead to conflicts over roles and responsibilities, increasing workplace stress [33]. Interdisciplinary friction can happen due to unclear professional roles and a lack of recognition of the specific contributions of occupational therapists [34]. In addition, other new management approaches such as NPM principles showed that the sole emphasis on efficiency and productivity can conflict with the core values of occupational therapy, as it shifts from quality care to meeting quantitative targets [24].

Furthermore, management styles and workplace policies play a crucial role in shaping occupational therapists' empowerment and autonomy, as well as their wellbeing and job satisfaction. There can be detrimental effects of inadequate supportive policies in specific settings, such as mental health, including the absence of social support and lack of flexible working hours and opportunities for professional development, contributing to turnover and workplace stress [27]. Lack of managerial support and clear policies can leave occupational therapists feeling undervalued and isolated, exacerbating workplace stress [28, 31]. In addition, top–down management styles are a significant barrier to professional autonomy and can further restrict occupational therapists' ability to adjust and engage effectively in their roles, leading to reduced effectiveness and increased stress [24, 31].

### 3.5. Advocacy and Strengthening Approaches

The studies together recommend some key strategies to strengthen the situation of occupational therapists within healthcare systems using an ecological system-level approach. These include further advocacy for the unique role of occupational therapy to enhance their visibility and impact [22], ongoing professional development to enhance evidence-based practices [27], developing personalized and targeted mentorship and supervision opportunities, building a strong professional identity to effectively navigate the complexities of healthcare environments [28], and enhancing career mobility through interprofessional collaboration [24].

Developing and maintaining an occupation-based focus in practice, with an emphasis on patient-centered care, were suggested as the key strategies to support professional resilience and identity and more importantly provide occupational therapists with a common language to explain interventions [22, 26]. In addition, implementing the right occupational models and assessment approaches within practice can assist them to maintain this occupation-based focus [26]. Practical, evidence-based guidelines are necessary to direct the practice, particularly with marginalized clients, to deliver optimal care during crisis periods. These can include clear job roles, enhancing workplace design, interprofessional cooperation, and providing more holistic and functional support systems to reduce stress and prevent burnout. Furthermore, creating an organizational culture with open communication and mutual continuous feedback can create a psychologically safe environment that enhances autonomy and belonging.

Interpersonal factors, both internal and external, including communication, networking, and negotiation were emphasized with a major impact on personalized care, career development [22], recognition, and professional identity [25, 33]. Interpersonal skills were suggested as key factors which moderate for job challenges while maintaining professional wellbeing [26]. They help with managing challenges, enhancing resilience, maintaining career longevity [22, 26], and reducing the sense of isolation [25]. Furthermore, to maintain career longevity, it is suggested that occupational therapists should be able to identify the “right” time to leave their organization, to enhance their career mobility opportunities, particularly if there is an imbalance of professional values within the organization [22].

Provision of effective and quality supervision, leadership style, and multidisciplinary team structures were highlighted by most papers, as key factors in improving professional development and enhancing career identity [22, 24–26, 28, 33]. The right supervision can mitigate workplace challenges by providing opportunities for reflective learning and a debriefing space [22, 26]. Junior occupational therapists valued frequent and structured mentorship/supervision opportunities for enhancing their personal and professional development [22, 24, 25, 33]. However, the more experienced and senior the occupational therapists become, the more flexibility and depth they may require in terms of more specialist external support [27]. Although the lack of availability of supervision can poorly influence job satisfaction [24], when clinicians are able to access and engage in quality supervision, it can be used as a strategy to develop professionally and maintain professional identity [22].

In addition, it is required to strike a balance between receiving supervision and being empowered to provide supervision to others and become a leader in the field. Overall, effective supervision and mentorship are highlighted for professional development, particularly providing direction and support for junior team members [25, 33], while also empowering experienced therapists to have opportunities for leadership and mentorship roles [27]. Quality supervision can provide opportunities for maintaining professional development and identity [26], as well as managing the emotional demands of the role.

In addition to supervision and leadership supports, sociocultural aspects of the society could impact career identity. For example, Jordanian occupational therapists highlighted that recognition, social support, and advocacy significantly influence their job satisfaction and career development. This suggests that cultural and social factors at the societal level play a crucial role in shaping occupational therapists' perceptions of their professional satisfaction and work environment [34].

## 4. Discussion

Job satisfaction is multifaceted and dynamic, impacted by various intrinsic and extrinsic factors. The interactions between these factors, and how they influence retention or attrition within an organization, can be complex. Job satisfaction has a significant direct or indirect impact on all the partners in various ways, including organizations, employees, consumers, and external stakeholders. Considering the significant role of job satisfaction on service quality, it is crucial to consider an ecological system-level approach to promote a positive working environment. This review highlights that although occupational therapists find their job rewarding mainly because of its humanistic aspect as well as the client-centeredness of the job, their job satisfaction is impacted by a range of factors, including autonomy, career identity, role diversity, problem-solving, and self-efficacy. Therapists also highlighted the disproportionate burden of administrative tasks that can impact the personalization and client-centered approaches to the service provision. In addition, they would like more collaboration and integration with other disciplines, as well as more empowering leadership and access to quality supervision, for professional growth and career mobility. Furthermore, enhancing professional resilience was highlighted, especially for those working with more specialized populations, such as those with mental illnesses and eating disorders [22, 26, 31].

Although occupational therapists expressed a general satisfaction with their work and believe in its value, they often feel unsatisfied with their professional profile, especially their career identity and public image. In addition, they feel there is a misalignment between the reductionist medical model, which focuses on clinical aspects and the biology of illness, compared with the more holistic, integrated and functional approaches of occupational therapists [35]. This is consistent with previous work indicating high level of administration tasks as a key barrier to practice [36]. Overall, occupational therapists feel that the status of their public profile is low, and they have been poorly understood by public, clients, and other disciplines. There has been a historic difficultly in defining the unique contribution of occupational therapy to healthcare and society. This can be due to lack of clear definition and recognition of the discipline, as well as lack of direction around job identity, which can impact their confidence as health professionals [35].

In order to enhance career identity and job satisfaction in occupational therapy, a critical examination of various factors across multiple levels is required. For example, although autonomy is frequently cited as a key factor, it needs to be critically paired with networking and career mobility to enrich professional identity in a meaningful way. The combination of autonomy alongside providing opportunities for connection and collaboration deepens practitioners' knowledge and skills and enhances motivation, engagement, resilience, and collaboration for more innovative approaches. In addition, it also assists in more recognition about the discipline, its unique contributions, and practical importance. However, this approach needs a strategic direction with a delicate balance. Solely increasing autonomy without considering the individual's capacity can result in mismatches between personal capabilities and job demands, potentially increasing stress rather than job satisfaction. Similarly, although career mobility can provide opportunities for professional growth and broader exposure, it should be managed in a way that does not impact the continuity of care or reduce professional focus. Furthermore, if there is a mismatch or lack of alignment between individuals' professional values and the organization values, even well-intentioned autonomy, career progression, and mobility initiatives might result in failure. Hence, planning for enhancing autonomy, networking, and career mobility needs to be aligned with the organizational vision and mission and integrated with the organizational strategies for sustainable impact in enhancing career identity and satisfaction [24].

Occupational therapists highlighted the role of leadership style, mentorship, and supervision in strengthening their job identity through personalized approaches that empower them to achieve their professional vision. Quality supervision opportunities were emphasized beyond tokenism approaches. This includes transformational leadership approaches, to inspire and empower individuals and encourage meaningful networking, connections, and capacity building [26].

Occupational therapists necessitate action for leadership changes, including advocating for the professional status of occupational therapy. Leaders are expected to develop a consistent leadership style including clear and open communication to help establish a psychologically safe environment where everyone feels supported, valued, and heard. In addition, transparent policies about professional development pathways, rewards (tangible and intangible), and equitable access to resources and opportunities are crucial to empower team members to thrive to their fullest potential. If the leadership approaches are integrated effectively, they can enormously enhance public and professional recognition of occupational therapy.

At the system level, clear guidelines and practical evidence–based frameworks and models around the discipline were suggested to enhance the confidence in the field [22]. An example is limited practical models for marginalized populations that require a more specific and personalized approach. Currently, the field is more theoretically based than empirical [35]. In addition, further funding and resources were suggested to advocate for the discipline [37] to enhance the research, evidence-based practices, and public image of occupational therapy. According to Moore et al. [28], limited resources can result in delayed staff replacement, retention issues, workload pressure, and compromised care standards. However, proactive planning such as workforce management and professional development can mitigate these challenges. Job satisfaction is directly impacted by the availability of resources, including meeting workforce demands and professional development through appropriate and timely recruitment [31], and access to appropriate physical spaces and tools required for intervention [34]. It was also highlighted in the most recent Victorian Occupational Therapy Workforce Report [38] that demand for occupational therapy services outweighs resources, which can lead to delays with recruitment, impacting retention and job satisfaction for an underresourced team [31]. This report and another report in Canada indicated that most occupational therapists were employed in public health settings, such as hospitals, residential care services, schools, and rehabilitation facilities, and only a small percentage work in private settings [39]. The changes at the system and policy levels are important, as they are directly impacting the guidelines and practices at the organizational as well as individual levels.

There are some indications based on the findings for improving the job satisfaction of occupational therapists. Occupational therapists have a functional and holistic approach to health and wellbeing and are a key source of motivation for empowering clients to take their health and wellbeing under control [35]. Occupational therapists consider physical, psychological, and social perspectives of health in a functional way, focusing on discovering the root causes of issues and not simply removing the symptoms. They strive for a personalized approach to care based on various contexts, including interconnected societal factors such as cultural, social, organizational, and physical variables [35]. For facilitating this process, they require a high level of networking and multidisciplinary collaboration, and this capability needs to be used in a diverse range of settings to assist with integrated care provision that improves various dimensions of quality of care [35]. Providing appropriate interprofessional training as well as adjusting the university curriculum can be fundamental to enhance the clarification about the field of occupational therapy and its specific nature. Incorporating communication skills, multidisciplinary approach to care, and resilience development are beneficial to enhance self-care adjustments, the required social changes, and advocacy, particularly in marginalized populations. This will empower occupational therapists to embrace client-centered models and occupational justice ideals for the provision of quality care [22, 26, 33, 35].

To provide direction for personal and professional development, it is important to develop career clarity and vision and provide a flexible working atmosphere that allows career advancement/planning and mobility. This will enhance the career identity for occupational therapists to be understood and accepted by other disciplines [27]. It is recommended to consider a balanced approach when recruiting new graduates and experienced team members, to complement each other and at the same time provide a mentoring atmosphere for personal and professional growth.

Further research can be beneficial in understanding the existing relationships between the occupational therapists' job satisfaction, perceptions of impacting factors, and their retention. In addition, more research can be beneficial in understanding the drivers of positive working environments and how they impact job satisfaction. Furthermore, considering that there is an increase in healthcare funding models, particularly the privatization of some of the sectors (e.g., disability and aged care), this shift can result in an increased demand for occupational therapists. However, it is unclear whether this translates to more satisfying opportunities within self-employed sectors, private and nonprofit settings, which may require further investigations.

### 4.1. Limitations

This review includes some limitations such as potential publication bias as two studies were published by the same authors, in the same setting, with the same participants [28, 31]. In addition, the review only included studies published in English [17]. Furthermore, the sample sizes in some of the studies were very small; examples are Bendixen and Ellegård [24], Ashby et al. [22], and Devery et al. [26] with 6, 9, and 10 participants, respectively, with the other studies including 14 or more participants. Finally, some specific terminologies were used such as “determinant∗,” “factor∗,” and “influence∗.” Although these keywords were selected to have a more specific focus on the relevant papers, some studies might have been missed which did not include these exact keywords in their titles and abstracts.

## Figures and Tables

**Table 1:** Databases and search terms used to identify literature for review.

**Database**	**Search terms**	**No. of articles**
*MEDLINE*	Ti (“occupational therapy” or “occupational therapist∗” or “ot”)	46
AND	Ti (MH (“job satisfaction” or “work satisfaction” or “employee satisfaction”))	
AND	“determinant∗” or “factor∗” or “influence∗”	
Limiters	English, scholarly (peer-reviewed) journals	
*CINAHL*	TI (“occupational therapy” or “occupational therapist∗” or “ot”)	18
AND	TI (“job satisfaction” or “work satisfaction” or “employee satisfaction”)	
AND	“determinant∗” or “factor∗” or “influence∗”	
Limiters	English, scholarly (peer-reviewed) journals	
*Scopus*	TITLE-ABS-KEY ((“occupational therapy”) OR (“occupational therapist∗”) OR (“ot”))	86
AND	TITLE-ABS-KEY ((“job satisfaction”) OR (“work satisfaction”) OR (“employee satisfaction”))	
AND	TITLE-ABS-KEY ((“determinant∗”) OR (“factor∗”) OR (“influence∗”))	
Limiters	English, Healthcare subject field	
*AMED*	TI (“occupational therapy” OR “occupational therapist∗” OR “ot”)	7
AND	TI (“job satisfaction” OR “work satisfaction” OR “employee satisfaction”)	
AND	(“determinant∗” OR “factor∗” OR “influence∗”)	
Limiters	English, peer reviewed	
Total records identified after database searching		157
Total records after duplicates removed		104

**Table 2:** Key methodological features and findings from reviewed studies.

**Author (year) country**	**Aims**	**Study design (qualitative research approach)**	**Sample/participants**	**Data collection and analysis**	**Results (key findings/themes)**	**Limitations**
Ashby et al. [22] Australia	Present strategies to sustain professional resilience, professional identity and occupation-based practice, using PRIOrity model	Narrative inquiry	9 OTs (2 male) with more than 2 years of experience in mental health and whom have worked in more than 1 workplace Mean experience was 14.3 years	2 phased interviews Ethics approved Purposive sampling Participant checking occurred Thematic analysis	• Job satisfaction: All participants found working with mental health clients satisfying. • Professional resilience: Central to occupational therapy, though challenging in mental health settings, it can be sustained by a strong professional identity. • Belief in occupational therapy: All participants affirmed the role of occupation in supporting client wellbeing. • Use of occupation-based models: These models supported professional resilience and provided a language for effective communication with clients and colleagues. • External theoretical pressure: Participants felt pressured to adopt theories from other disciplines perceived as more evidence based. Occupational therapists (OTs) face pressure to integrate more medically and scientifically rigorous methods that emphasize measurable outcomes, creating tension with their traditional, holistic approaches that focus on patients' overall wellbeing. This challenge risks compromising the core values of occupational therapy, which prioritize a comprehensive understanding of health including physical, emotional, and social aspects. • Professional socialization: Crucial for maintaining professional resilience, identity, and supporting retention and recruitment. • Effective professional supervision: Maintains professional identity, enhances clinical skills, and is more beneficial than generic supervision from another discipline. • Professional mobility: Strategic movements between roles or settings sustain professional resilience. • Advocacy for occupation-based practice: Essential for articulating the value of occupational therapy in multidisciplinary environments. • Professional bilingualism: Necessary to communicate effectively across disciplines, enhancing collaboration. • Pressure and advocacy: Navigating external pressures while advocating for occupation-based practice is crucial for maintaining professional integrity.	• Small sample size • Participants from one organization, working only in mental health

Bendixen and Ellegard [24] Denmark	Investigate OTs' job satisfaction under a changing regime focusing on therapists' everyday lives In 2006, CUHG merged its occupational therapy and physiotherapy departments, adopting the holistic International Classification of Functioning, Disability, and Health (ICF) paradigms focused on meaningful occupations over traditional symptom-based treatments. Despite the progressive shift, the change prompted staff unease, especially in orthopedics, which retained a more traditional focus. To navigate these challenges, the department implemented a time–geography approach, aiming to smooth the transition and encourage staff adaptation through enhanced engagement with new practices	Mixed method–sequential transformative design	OTs working in ORT unit in Copenhagen, Denmark 9 OTs completed diary component of research 6 of those OTs completed the in-depth interview Ethics obtained	Time–geographic diary approach Data collection by semistructured in-depth interviews Interviews analyzed by meaning condensation and conceptual interpretation Member checking occurred	• Workplace structure: The new layout intended to integrate OTs and physiotherapists impedes interaction and supervision, reducing effectiveness. • Management style: A top–down approach restricts autonomy, potentially leading to poor performance and increased resignations. • Handling interruptions: Experienced OTs manage interruptions effectively, enhancing workflow; however, newcomers find these disruptions challenging, impacting their ability to supervise effectively. • Job characterization by interruptions: Interruptions are common and expected in OT roles, but their management varies by experience level. • Time management in work projects: OTs are surprised by the skewed allocation of time favoring general tasks over patient-centered activities. • Attitudes towards new paradigms: The shift towards a more holistic clinical practice garners mixed reactions; experienced OTs view it as a growth opportunity, while newcomers feel apprehensive. • Professional autonomy and decision-making: Autonomy varies significantly among OTs; some feel empowered, others constrained by management's decisions. • Influence of new public management (NPM): NPM principles have introduced authority constraints that affect both clinical and administrative duties of OTs. • Implementation of new OT paradigms: There are diverse reactions to the shift towards holistic, activity-based practices, reflecting varying levels of acceptance among OTs. • Cultural clashes and mistrust: New paradigms lead to cultural clashes within the department, highlighting the need for better knowledge sharing and change management. • Departmental design flaws: The intended collaborative space between OTs and physiotherapists fails, hindering effective supervision and interaction due to poor spatial configuration.	• Specific to local department change • Researcher was responsible for local department change (researcher bias) • Small sample size

Wesley & Clemson [25] Australia	Determine issues of job satisfaction and dissatisfaction in an OT department	Exploratory qualitative study	26 OTs from one hospital OT department Purposive sampling, random allocation to one of 4 focus groups • 5 new graduates • 11 OTs less than 25 years old	Focus group Discussions moderated based on Herzberg's motivator/hygiene factors and Bordeiri's adaptions Data double coded and thematically analyzed using Herzberg's 5 motivator and 5 hygiene factors and Bordieri's support for training factor Member checking and revision of coding occurred	• Interpersonal relationships: Strong interpersonal relationships with coworkers and patients are a significant source of job satisfaction. • Professional autonomy: High value is placed on professional autonomy and the ability to influence department decisions and set job standards. • Recognition and achievement: Recognition for work done and personal achievements contributes positively to job satisfaction. • Support and supervision: Availability of support and supervision from managers and senior staff plays a crucial role in job satisfaction, especially for less-experienced therapists. • Work conditions and environment: Physical work environment and conditions such as space and administrative support impact job satisfaction. • Training and development opportunities: Opportunities for further education and training are essential for job satisfaction but are often limited by budget constraints. • Management and administrative support: Effective management and clear administrative policies are important to job satisfaction but often need improvement. • Salary and advancement: Competitive salary and opportunities for career advancement are less commonly cited but still relevant to overall job satisfaction. • Work–life balance and caseload management: Managing fluctuating caseloads and maintaining work–life balance are critical for preventing burnout and enhancing job satisfaction. • Role clarity and expectations: Clear role definitions and expectations help in reducing job dissatisfaction and improving staff morale.	• Older study • Not all participants comfortable to freely express their opinions in a focus group • Many participants were junior therapists • Participants from one organization

Devery, Scanlan and Ross [26] Australia	Explore the relationship between professional identity, job challenges, burnout, and job satisfaction of OTs working in eating disorder units	Mixed methods–explanatory sequential design	10 OTs (8 completed interviews), registered in Australia with current or recent employment in an eating disorder unit Sample obtained through existing networks	Online survey followed by semistructured interview by phone Thematic analysis of interview data Member checking occurred	• Professional identity challenges: Therapists face challenges in defining and maintaining their professional roles within eating disorder services. • Risk of burnout: High burnout rates among therapists due to intense emotional and physical job demands. • Low job satisfaction: Job satisfaction is compromised by structural limitations and the emotional intensity of the work. • Structural job challenges: Issues such as unclear role definitions, inadequate funding, and lack of specialist supervision. • Personal job challenges: Feelings of being underprepared and a general lack of supportive research for therapeutic practices. • Client-related challenges: Difficulties managing countertransference, high relapse rates, comorbidities, and increased risk factors among clients. • Professional wellbeing maintenance: Strategies and practices implemented by therapists to sustain their professional and personal wellbeing. • Professional satisfaction: Therapists find client interactions rewarding, contributing positively to job satisfaction. • Occupation focus maintenance: Emphasis on occupation-based practices valued by both clients and therapists for effective treatment outcomes. • Supervision access: Regular access to professional supervision helps mitigate the challenges of the job. • Special interest group participation: Joining special interest groups boosts therapists' confidence and professional network. • Work–life balance importance: Maintaining a balance between personal life and work is crucial for therapist wellbeing. • Research and evidence needs: There is a critical need for more research to develop a solid evidence base for occupational therapy practices in eating disorders, aiming to enhance therapeutic effectiveness and professional validation.	• Small sample size (representative of employment of OT in this field) • Niche OT field therefore results may not be transferrable

Hayes et al. [27] Australia	Determine factors which influence retention and perceptions of supervision and professional development	Exploratory survey	57 OTs working in North West Mental Health service (Melbourne, Victoria) include community-based and acute inpatient care 53 females, 4 males	Survey with closed and open-ended questions Open-ended question focus: Supervision satisfaction and focus, constraints with receipt of training Thematic analysis of open-ended questions	• Recruitment challenges: Difficulty in attracting new occupational therapists to mental health due to perceived job demands and role ambiguity. • Retention difficulties: High turnover and difficulty retaining experienced therapists, impacted by job satisfaction and work environment factors. • Role definition: Lack of clear role definition and concerns about the generic nature of work, reducing job satisfaction and professional identity. • Professional identity: Challenges in maintaining a strong professional identity amidst high levels of role blurring with other professions. • Stress and burnout: High levels of job-related stress and burnout, influencing both recruitment and retention negatively. • Supervision quality: The quality and frequency of professional supervision available, affecting job satisfaction and professional growth. • Career advancement: Limited opportunities for career progression within the mental health sector, impacting long-term retention. • Training and development: Need for more discipline-specific continuing professional development to enhance skills and job satisfaction. • Interprofessional Collaboration: The need for improved collaboration and training with other healthcare professionals to enhance service delivery and professional respect. • Organizational support: The importance of organizational support, including better policies for client referral to occupational therapy, to enhance job satisfaction and retention.	• Depth of information limited with use of questionnaire for open-ended questions • Job satisfaction not a major focus • Participants from one organization, working only in mental health

Moore et al. [28] Australia	Determine the influence of managers on the job satisfaction of OTs	Hermeneutical phenomenological	14 female participants working as OTs in public sector of Greater Metropolitan Sydney 11 working in teaching/district hospital 3 in MDT community teams Purposive sampling Ethics obtained	Moustakas (1994) key principles applied to guide data collection, analysis, and interpretation Data collected from semistructured interviews Analysis followed Colaizzi's (1978) 6 steps for phenomenological data analysis and interpretation Data saturation reached Member checking occurred	• Approachability: Managers who are accessible and supportive enhance team morale and job satisfaction. • Consistent leadership: Stability in management behavior fosters a trustworthy and fair work environment. • Career development support: Active encouragement and support in skill development boost employee motivation and satisfaction. • Transparent communication: Open and inclusive communication from managers keeps teams well informed and engaged. • Clinical engagement: Managers who participate in clinical activities gain respect and enhance team dynamics. • Advocacy: Strong representation for the department's needs by managers promotes a supportive workplace. • Fair policy implementation: Clear and equitable policies from managers contribute to organizational integrity and staff satisfaction. • Intimidation by management: Unapproachable managers who instill fear create a toxic work environment. • Management inconsistency: Fluctuating moods and unpredictable decisions by managers lead to employee insecurity and dissatisfaction. • Professional boundaries: Managers who allow personal relationships to influence decisions disrupt workplace fairness. • Confidentiality issues: Breaches in confidentiality by managers undermine trust and professional relationships. • Favoritism: Preferential treatment and bias in managerial decisions erode team morale and equality.	• Specific focus area of effect of manager on job satisfaction phenomenon

Moore et al. [31] Australia	Describe the factors affecting job satisfaction and dissatisfaction in a sample of OTs working in public health in NSW, Australia	Hermeneutical phenomenological	14 female participants working as OTs in public sector of Greater Metropolitan Sydney 11 working in teaching/district hospital, 3 in MDT community teams Purposive sampling Range of experience from 6 months to 20 years Ethics obtained	Data collection by semistructured interviews based on Herzberg's Motivation–hygiene theory Thematic analysis Data saturation achieved Member checking occurred	• Career fulfillment: Occupational therapists find their careers rewarding due to diverse roles and significant impacts on clients' lives. • Diversity in work: Job satisfaction is bolstered by the variety within roles, including different specializations and daily tasks. • Client relationships: Positive relationships with clients enhance job satisfaction. • Achievement: Therapists derive satisfaction from achieving therapeutic goals with clients. • Autonomy: Therapists appreciate the autonomy to plan and manage their treatment sessions and workdays. • Lack of Professional Recognition: Occupational therapists often feel undervalued due to poor recognition and understanding from colleagues and the public. • Role Misunderstanding: The occupational therapy profession often suffers from poor role definition and low status. • Resource Limitations: Limited healthcare budgets result in fewer resources for OT departments, contributing to dissatisfaction. • Impact of Inadequate Resources: Inadequate resources negatively affect the quality of care and job satisfaction.	

Oven & Domajnko [32] Slovenia	Job satisfaction and creativity at work among occupational therapy practitioners: A mixed-method study	Mixed methods	21 females, 1 male Mean age of 44 with average experience of 19 years Mixed areas of work, largest cohort in mental health and rehabilitation	22 OTs engaged in a focus group which was the exploratory phase and guided the development of the quantitative survey	• Most of the participants agreed or strongly agreed they were satisfied at work. • Main themes emerged relating to creativity and work satisfaction: Uniqueness of each client case; personal characteristics; work experience; work environment; relationships with colleagues; relationships with superiors • Creativity positively impacted both life and work satisfaction of OTs • Creativity is key: Occupational therapists thrive in creative roles, boosting job satisfaction significantly. • Empowerment matters: Autonomy in the workplace fuels creativity more than any other factor. • Routine is the enemy: Routine tasks dampen creativity and reduce job satisfaction. • Growth inspires: Personal and professional development opportunities are crucial for fostering creativity. • Feedback fuels creativity: Positive feedback from peers and clients enhances motivation and creativity. • Supportive environment: A supportive work environment enhances creativity, though less so than autonomy.	• Risk of moderator bias with a focus group

Shiri [33] New Zealand	Explore the factors influencing job satisfaction of OTs working in acute care	Interpretive inquiry	20 physical acute care OTs practicing on North Island (F: 18, M: 2) Clinical experience range: 1–3 years (*N* = 18) and 4+ years (*N* = 2) Purposive sampling Ethics obtained	4 focus groups Thematic analysis Data and investigation triangulation employed Member checking resulted in 99% positively agreed with findings Data saturation attained	• Supportive team dynamics: Satisfaction is enhanced by effective collaboration and support from multidisciplinary team members, including other occupational therapists. • Valued supervision: Occupational therapists appreciate structured and frequent supervision, which contributes to their professional growth and job satisfaction. • Professional recognition: Being recognized and understood by other team members, particularly in a supportive multidisciplinary environment, is crucial. • Role misunderstanding: There is a significant lack of understanding of the occupational therapist's role, especially by doctors, which leads to dissatisfaction. • Professional status and boundaries: Occupational therapists feel their professional status is not on par with physiotherapists, leading to issues with practice boundaries and respect within the healthcare team. • Communication barriers: Poor communication, particularly with doctors regarding patient care, frustrates occupational therapists and affects their ability to perform effectively. • Distinctive role definition: Use of specific occupational therapy theoretical models, assessments, and treatment approaches to clearly differentiate and strengthen the OT role. • Educational initiatives: Implement formal education processes with multidisciplinary teams to enhance understanding and clarification of the OT role. • Proactive documentation: Initiate the use of incident forms for patients who have not been assessed by an occupational therapist prior to discharge to highlight the necessity of OT involvement and ensure visibility.	• Not all participants comfortable to freely express their opinions in a focus group • Most participants were junior therapists • Participants from acute care field only

Tariah et al. [34] Jordan	Explore factors influencing job satisfaction and dissatisfaction among Jordanian OTs	Explorative qualitative	93 OTs (F: 57, M: 36), 80% younger than 30 years, 85% had less than 5 years' experience Experience ranged from minimum of 6 months to 15+ years Range of workplace settings, majority in hospitals (42.6%) and pediatrics (32.3%) Solely academic OTs were excluded Purposive sampling Ethics obtained	Data collected by structured open-ended survey questions Inductive content analysis performed and then thematic analysis Member checking occurred with 5 participants	• Humanistic fulfillment: Occupational therapists derive satisfaction from the human-centered nature of their work, which focuses on helping clients achieve better quality of life. • Professional role satisfaction: Therapists appreciate the positive aspects of their professional duties and the impact they have on clients, enhancing job satisfaction. • Benefits and development: Job security and opportunities for professional development are valued as significant contributors to occupational satisfaction. • Supportive work environment: A positive and supportive workplace is crucial for enhancing the satisfaction levels of occupational therapists. • Resource constraints: Limited support and inadequate resources within the healthcare system are major sources of dissatisfaction. • Financial and recognition issues: Inadequate financial rewards and lack of professional recognition within the healthcare sector led to job dissatisfaction. • Role awareness: The lack of public and professional understanding of the occupational therapy role negatively impacts job satisfaction. • Systemic improvements: There is a strong call for improvements in professional recognition, resource allocation, and support systems. • Collaborative efforts: Enhanced cooperation among occupational therapists, healthcare administrators, and policymakers is necessary to improve working conditions. • Setting-specific satisfaction: Job satisfaction varies significantly with the setting, with therapists in school-based settings generally more satisfied due to different work dynamics and client interactions. • Client interaction: Direct interaction with clients is a major positive factor influencing job satisfaction among occupational therapists. • Workload stress: Heavy caseloads in certain settings, such as schools, are identified as significant stressors that affect job satisfaction. • Research gap: There is a noted lack of research on occupational therapy in developing countries, highlighting a need for more targeted studies in these regions. • Extrinsic vs. intrinsic factors: Variations in job satisfaction are influenced by both extrinsic factors like pay and intrinsic factors like personal fulfillment from work achievements.	• Depth of information limited with use of questionnaire for open-ended questions • Majority of participants were junior therapists • OT considered new profession in Jordan

Abbreviations: F, female; M, male; MDT, multidisciplinary team; NSW, New South Wales; ORT, orthopedic subdepartment; OT, occupational therapist.

## Data Availability

All data supporting this study is included in the article.
